# Metal-Based Biologically Active Compounds: Synthesis, Spectral, and Antimicrobial Studies of Cobalt, Nickel, Copper, and Zinc Complexes of Triazole-Derived Schiff Bases

**DOI:** 10.1155/2011/901716

**Published:** 2011-12-19

**Authors:** Kiran Singh, Yogender Kumar, Parvesh Puri, Chetan Sharma, Kamal Rai Aneja

**Affiliations:** ^1^Department of Chemistry, Kurukshetra University, Kurukshetra 136 119, India; ^2^Department of Microbiology, Kurukshetra University, Kurukshetra 136 119, India

## Abstract

A series of cobalt, nickel, copper, and zinc complexes of bidentate Schiff bases derived from the condensation reaction of 4-amino-5-mercapto-3-methyl/ethyl-1,2,4-triazole with 2,4-dichlorobenzaldehyde were synthesized and tested as antimicrobial agents. The synthesized Schiff bases and their metal complexes were characterized with the aid of elemental analyses, magnetic moment measurements, spectroscopic and thermogravimetric techniques. The presence of coordinated water in metal complexes was supported by infrared and thermal gravimetric studies. A square planar geometry was suggested for Cu(II) and octahedral geometry proposed for Co(II), Ni(II), and Zn(II) complexes. The Schiff bases and their metal complexes have been screened for antibacterial (*Pseudomonas aeruginosa, Bacillus subtilis*) and antifungal activities (*Aspergillus niger, A. flavus*). The metal complexes exhibited significantly enhanced antibacterial and antifungal activity as compared to their simple Schiff bases.

## 1. Introduction

Of late due to the constant emergence of antibiotics resistance to clinically used compounds, it is the need of the hour to develop novel antibiotics classes, which eventually would target the lipoid layer of the organisms and other aspects of pathogens' life cycle. Metal complexes may be subjected for the design and synthesis of such possibilities having such biological activities [1–3]. The chemistry of Schiff bases and their structural analogues has occupied a place of considerable importance [4] as they easily form stable complexes with most transition metal ions [5, 6] and well-established biological properties. 1,2,4-triazoles nucleus and their derivatives emerge rapidly with the advances of modern heterocyclic chemistry, promising a variety of medical applications such as antibacterial, antifungal, anticancer, antitumor, anticonvulsant, anti-inflammatory, and analgesic properties [7–12]. Schiff bases of 1,2,4-triazoles find diverse applications and extensive biological activity. The incorporation of the 1,2,4-triazole unit into Schiff base macrocycles is of considerable current interest as complexes of 1,2,4-triazoles are being developed for potential use in applications such as magnetic materials and photo chemically driven molecular devices [13]. Schiff bases derived from 3-substituted-4-amino-5-mercapto-1,2,4-triazoles show analgesic, antimicrobial, anti-inflammatory, and antidepressant activities [14]. 

The aim of the present study was to modify the bioactivities of 1,2,4-triazole Schiff bases and gain the relative derivatives with better curing effect, optimization of hydrophilic/lipophilic character, and improve the bioavailability by coordinating them with transition metal ions. During the course of this work, a series of cobalt, nickel, copper, and zinc complexes of bidentate Schiff bases derived from the condensation reaction of 4-amino-5-mercapto-3-methyl/ethyl-1, 2, 4-triazole with 2, 4-dichlorobenzaldehyde have been synthesized. The synthesized Schiff bases and their metal complexes were characterized with the aid of elemental analyses, magnetic moment measurements, spectroscopic, and thermogravimetric techniques. The Schiff bases and their metal complexes have been screened for antibacterial (*Pseudomonas aeruginosa*, *Bacillus subtilis*) and antifungal activities (*Aspergillus niger*, *A. flavus*).

## 2. Experimental

### 2.1. Materials and Methods

All the chemicals used in the present investigation were of Analytical grade and used without further purification. The metal contents were determined using standard gravimetric methods; cobalt was estimated as cobalt pyridine thiocyanate, nickel as nickel dimethylglyoximate, copper as cuprous thiocyanate, and zinc as zinc ammonium phosphate [15]. 

The Perfit electrical melting point apparatus is used to record melting points of the synthesized complexes and are uncorrected. Carbon, hydrogen, and nitrogen were estimated using Perkin-Elmer 2400 Elemental Analyzer at Punjab University, Chandigarh. Electronic spectra of metal complexes were recorded in DMF on a Systronics 2203 double-beam spectrophotometer in the region 1100–200 nm. IR spectra were recorded on a MB-3000 ABB spectrometer in KBr/Nujol mulls in the range 4000–250 cm^−1^. Proton NMR spectra were recorded in DMSO-d_6_ on a Bruker ACF 300 spectrometer at 300 MHz using “tetramethyl silane” as the internal standard. Magnetic moments were measured at Institute Instrumentation Centre, IIT Roorkee on vibrating sample magnetometer (Model 155). The Perkin Elmer (Pyris Diamond) instrument was used to carry out thermal analysis of metal complexes in atmospheric air at the heating rate of 10°C Min^−1^ using a reference to alumina powder. The EPR spectrum of the copper(II) complex was recorded at SAIF, IIT, Bombay, using VARIAN E-112 X-band EPR spectrometer with cylindrical quartz sample tube operating at microwave frequency 9.5 GHz. Field calibration was checked using tetracyanoethylene (TCNE) free radical for which *g* = 2.0027 at room temperature.

### 2.2. Syntheses

4-Amino-5-mercapto-3-methyl-1,2,4-triazole (*ammt*) and 4-amino-3-ethyl-5-mercapto-1,2,4-triazole (*aemt*) were prepared by reported literature method [16].

#### 2.2.1. 4-[(2,4-Dichloro-benzylidene)-amino]-5-mercapto-3-methyl-1,2,4-triazole (HL^1^)

**Figure 1:**
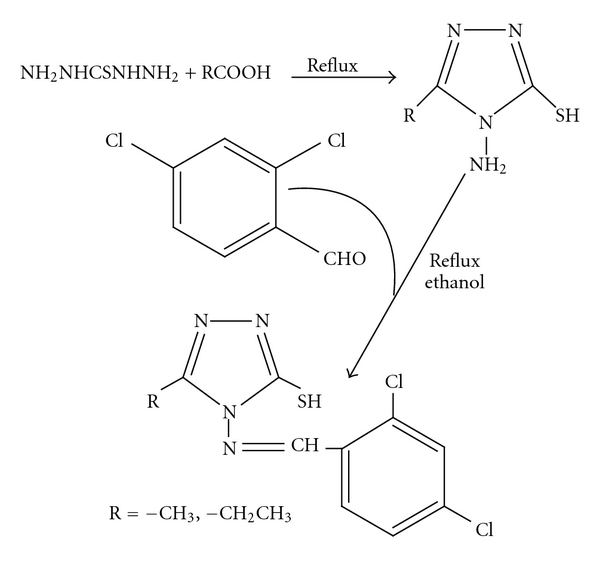
Scheme for the synthesis of Schiff bases.

An ethanolic solution (40 mL) of *ammt* (1.08 g, 8.33 mmol) was treated with 2,4-dichlorobenzaldehyde (1.45 g, 8.33 mmol). The reaction mixture was refluxed for 3 h, and then the clear solution was allowed to cool to room temperature. The solid material formed was removed by filtration, washed with cold ethanol, and recrystallized from ethanol and dried (Figure 1). 

m.p. 249–252°C, (found: C, 41.78; H, 2.78; N, 19.46%. Calcd. For C_10_H_8_Cl_2_N_4_S: C, 41.82; H, 2.81; N, 19.51%); MS: m/z (M + 1) 286.9, (M − 1) 284.9.

#### 2.2.2. 4-[(2,4-Dichloro-benzylidene)-amino]-3-ethyl-5-mercapto-1,2,4-triazole (HL^2^)

An ethanolic solution of *aemt* (1.41 g, 9.79 mmol) was added with stirring to an ethanolic solution of 5-nitro-furfuraldehyde (1.71 g, 9.79 mmol) and refluxed for 3 h, and the product was filtered off, washed with ice cold ethanol, and a light yellow crystalline product was obtained after recrystallization from ethanol (Figure 1). 

m.p. 236–239°C, (found: C, 43.22; H, 3.28; N, 18.43%. calcd. For C_11_H_10_Cl_2_N_4_S: C, 43.86; H, 3.35; N, 18.60%); MS: m/z (M + 1) 300.9, (M − 1) 298.9.

#### 2.2.3. Syntheses of Metal Complexes of [HL^1^] (1 : 1)

The hot ethanolic solutions of the HL^1^ (0.22 g, 0.76 mmol) were slowly added to the aqueous ethanolic solutions of acetates of Co(II) (0.19 g, 0.76 mmol), Ni(II) (0.19 g, 0.76 mmol), Cu(II) (0.15 g, 0.76 mmol), and Zn(II) (0.16 g, 0.76 mmol), which resulted in the immediate precipitation of metal derivatives. The products formed were filtered and purified by washing thoroughly with warm water, with aqueous ethanol to remove unreacted metal acetates or ligands, and finally with acetone and dried.

Co(L^1^)OAc·3H_2_O: (found: C, 31.28; H, 3.38; N, 12.10; Co, 12.78% calcd. For C_12_H_16_Cl_2_CoN_4_O_5_S: C, 31.46; H, 3.52; N, 12.23; Co, 12.86%).

Ni(L^1^)OAc·3H_2_O: (found: C, 31.38; H, 3.43; N, 12.16; Ni, 12.72% calcd. For C_12_H_16_Cl_2_N_4_NiO_5_S: C, 31.47; H, 3.52; N, 12.23; Ni, 12.82%).

Cu(L^1^)OAc·H_2_O: (found: C, 39.44; H, 2.68; N, 16.88; Cu, 9.54% calcd. For C_12_H_12_Cl_2_CuN_4_O_3_S: C, 39.80; H, 2.73; N, 16.88; Cu, 9.57%).

Zn(L^1^)OAc·3H_2_O: (found: C, 30.99; H, 3.41; N, 11.96; Zn, 14.00% calcd. For C_12_H_16_Cl_2_N_4_O_5_SZn: C, 31.02; H, 3.47; N, 12.06; Zn, 14.07%).

#### 2.2.4. Syntheses of Metal Complexes of [HL^1^] (1 : 2)

The aqueous ethanolic solutions of acetates of Co(II) (0.17 g, 0.69 mmol), Ni(II) (0.17 g, 0.69 mmol), Cu(II) (0.14 g, 0.69 mmol) and Zn(II) (0.15 g, 0.69 mmol) were treated with the hot ethanolic solutions of the HL^1^ (0.40 g, 1.39 mmol). The products formed were filtered and purified by washing thoroughly with warm water, with aqueous ethanol to remove unreacted metal acetates or ligands and finally with acetone and dried.

Co(L^1^)_2_·2H_2_O: (found: C, 35.88; H, 2.67; N, 16.73; Co, 8.80% calcd. For C_20_H_18_Cl_4_CoN_8_O_2_S_2_: C, 36.00; H, 2.72; N, 16.79; Co, 8.83%).

Ni(L^1^)_2_·2H_2_O: (found: C, 36.00; H, 2.62; N, 16.55; Ni, 8.76% calcd. For C_20_H_18_Cl_4_N_8_NiO_2_S_2_: C, 36.01; H, 2.72; N, 16.80; Ni, 8.80%).

Cu(L^1^)_2_: (found: C, 33.65; H, 2.77; N, 13.61; Cu, 14.77% calcd. For C_20_H_14_Cl_4_CuN_8_S_2_: C, 33.77; H, 2.83; N, 13.63; Cu, 14.89%).

Zn(L^1^)_2_·2H_2_O: (found: C, 35.60; H, 2.61; N, 16.58; Zn, 9.68% calcd. For C_20_H_18_Cl_4_N_8_O_2_S_2_Zn: C, 35.65; H, 2.69; N, 16.63; Zn, 9.71%).

#### 2.2.5. Syntheses of Metal Complexes of [HL^2^] (1 : 1)

The hot ethanolic solutions of the HL^2^ (0.31 g, 1 mmol) were added to the aqueous ethanolic solutions of acetates of Co(II) (0.25 g, 1 mmol), Ni(II) (0.25 g, 1 mmol), Cu(II) (0.20 g, 1 mmol) and Zn(II) (0.22 g, 1 mmol), which resulted in the immediate precipitation of metal derivatives. The products formed were filtered and purified by washing thoroughly with warm water, with aqueous ethanol to remove unreacted metal acetates or ligands and finally with acetone and dried.

Co(L^2^)OAc·3H_2_O: (found: C, 33.00; H, 3.84; N, 11.77; Co, 12.43% calcd. For C_13_H_18_Cl_2_CoN_4_O_5_S: C, 33.07; H, 3.84; N, 11.86; Co, 12.48%).

Ni(L^2^)OAc·3H_2_O: (found: C, 32.97; H, 3.81; N, 11.81; Ni, 12.44% calcd. For C_13_H_18_Cl_2_N_4_NiO_5_S: C, 33.08; H, 3.84; N, 11.87; Ni, 12.44%).

Cu(L^2^)OAc·H_2_O: (found: C, 35.42; H, 3.18; N, 12.20; Cu, 14.48% calcd. For C_13_H_14_Cl_2_CuN_4_O_3_S: C, 35.42; H, 3.20; N, 12.71; Cu, 14.42%).

Zn(L^2^)OAc·3H_2_O: (found: C, 32.60; H, 3.78; N, 15.73; Zn, 9.31% calcd. For C_13_H_18_Cl_2_N_4_O_5_SZn: C, 32.62; H, 3.79; N, 15.97; Zn, 9.32%).

#### 2.2.6. Syntheses of Metal Complexes of [HL^2^] (1 : 2)

The aqueous ethanolic solutions of acetates of Co(II) (0.20 g, 0.83 mmol), Ni(II) (0.20 g, 0.83 mmol), Cu(II) (0.16 g, 0.83 mmol), and Zn(II) (0.18 g, 0.83 mmol) were treated with the hot ethanolic solutions of the HL^2^ (0.50 g, 1.6 mmol). The products formed were filtered and purified by washing thoroughly with warm water, with aqueous ethanol to remove unreacted metal acetates or ligands, and finally with acetone and dried.

Co(L^2^)_2_·2H_2_O: (found: C, 37.77; H, 3.18; N, 16.13; Co, 8.48% calcd. For C_22_H_22_Cl_4_CoN_8_O_2_S_2_: C, 38.00; H, 3.19; N, 16.12; Co, 8.48%).

Ni(L^2^)_2_·2H_2_O: (found: C, 38.80; H, 3.15; N, 16.14; Ni, 8.45% calcd. For C_22_H_22_Cl_4_N_8_NiO_2_S_2_: C, 38.81; H, 3.19; N, 16.12; Ni, 8.44%).

Cu(L^2^)_2_: (found: C, 39.21; H, 2.88; N, 16.83; Cu, 9.55% calcd. For C_22_H_18_Cl_4_CuN_8_S_2_: C, 39.80; H, 2.73; N, 16.88; Cu, 9.57%).

Zn(L^2^)_2_·2H_2_O: (found: C, 37.66; H, 3.15; N, 15.95; Zn, 9.32% calcd. For C_22_H_22_Cl_4_N_8_O_2_S_2_Zn: C, 37.65; H, 3.16; N, 15.97; Zn, 9.31%).

### 2.3. Antimicrobial Assay

#### 2.3.1. Test Microorganisms

Four microbial strains were selected on the basis of their clinical importance in causing diseases in humans. Two bacteria (*Staphylococcus aureus *MTCC 96 and *Bacillus subtilis *MTCC 121) and two fungi, *Aspergillus niger *and* A. flavus*, the ear pathogens isolated from the patients of Kurukshetra [17], were used in the present study for evaluation of antimicrobial activity of the synthesized compounds. All the bacterial cultures were procured from Microbial Type Culture Collection (MTCC), IMTECH, Chandigarh. The bacteria were subcultured on Nutrient agar, whereas fungi on Sabouraud's dextrose agar.

#### 2.3.2. Antibacterial Activity

The antibacterial activity of all the synthesized complexes was evaluated by using agar well-diffusion method. All the microbial cultures were adjusted to 0.5 McFarland standard, which is visually comparable to a microbial suspension of approximately 1.5 × 10^8^ cfu/mL. 20 mL of Mueller Hinton agar medium was poured into each Petri plate and plates were swabbed with 100 *μ*L inocula of the test microorganisms and kept for 15 min for adsorption. Using sterile cork borer of 8 mm diameter, wells were bored into the seeded agar plates, and these were loaded with a 100 *μ*L volume with concentration of 4.0 mg/mL of each compound reconstituted in the dimethylsulphoxide (DMSO). All the plates were incubated at 37°C for 24 hrs. Antibacterial activity of each compound was evaluated by measuring the zone of growth inhibition against the test organisms with zone reader (HiAntibiotic zone scale). DMSO was used as a negative control whereas Ciprofloxacin was used as positive control. This procedure was performed in three replicate plates for each organism [18, 19].

#### 2.3.3. Minimum Inhibitory Concentration (MIC) Determination

MIC of all the compounds against bacterial strains was tested through a modified agar well-diffusion method [20]. In this method, a twofold serial dilution of each compound was prepared by first reconstituting the compound in DMSO followed by dilution in sterile distilled water to achieve a decreasing concentration range of 256 to 0.5 *μ*g/mL. A 100 *μ*L volume of each dilution was introduced into wells (in triplicate) in the agar plates already seeded with 100 *μ*L of standardized inoculums (10^6^ cfu/mL) of the test microbial strain. All test plates were incubated aerobically at 37°C for 24 hrs and observed for the inhibition zones. MIC, taken as the lowest concentration of the chemical compound that completely inhibited the growth of the microbe, showed by a clear zone of inhibition, was recorded for each test organism. Ciprofloxacin was used as positive control.

#### 2.3.4. Antifungal Activity

The antifungal activity of Schiff bases and their metal complexes was evaluated by poison food technique [21]. The molds were grown on Sabouraud's dextrose agar (SDA) at 25°C for 7 days and used as inocula. The 15 mL of molten SDA (45°C) was poisoned by the addition of 100 *μ*L volume of each compound having concentration of 4.0 mg/mL reconstituted in DMSO, poured into a sterile Petri plate, and allowed it to solidify at room temperature. The solidified poisoned agar plates were inoculated at the center with fungal plugs (8 mm diameter) obtained from the colony margins and incubated at 25°C for 7 days. DMSO was used as the negative control whereas fluconazole was used as the positive control. The experiments were performed in triplicates. Diameter of fungal colonies was measured and expressed as percent mycelial inhibition.

Percent inhibition of mycelial growth = (dc − dt)/dc × 100.

dc: average diameter of fungal colony in negative control sets.

dt: average diameter fungal colony in experimental sets.

## 3. Results and Discussion

The Schiff bases (Figure 1) were soluble in ethanol and methanol. All the metal complexes are colored, nonhygroscopic solids stable in air, insoluble in water and many common organic solvents but soluble in DMF and DMSO, decomposed at higher temperature. The elemental analysis supports the formation of 1 : 1 and 1 : 2 metal complexes of HL^1-2^ with Co(II), Ni(II), Cu(II), and Zn(II) metal ions. The molar conductance values of the complexes (measured in 10^−3^ M DMF) are in the range 1.5–3.6 ohm^−1^ cm^2^ mol^−1^ indicating the nonelectrolytic nature [22]. The purity of ligands and their metal complexes has been checked by TLC.

### 3.1. IR Spectra

The IR spectral data of the Schiff base ligands and their complexes are listed in Table 1. The IR spectra of the complexes have been compared with those of the free ligand in order to determine the coordination sites that may get involved in chelation. By comparing, it was found that azomethine group frequency *ν*(N=CH) is present in the free ligands at 1595–1597 cm^−1^. This band is shifted to the lower frequency (22–24) by 5–15 cm^−1^ in the spectra of the complexes, indicating coordination of azomethine nitrogen towards the metal ion (M–N).

A new band at 485–514 cm^−1^ due to *ν*(M–N) is further confirmed the coordination of metal to azomethine nitrogen. The Schiff bases exhibit a weak broad band around ~2700 cm^−1^ due to *ν*(S–H) vibrations [23, 24]. This band disappeared in the spectra of the metal complexes indicating deprotonation and complexation through sulphur. In the spectra of metal complexes, bands appeared at 733–771 cm^−1^ and 334–352 cm^−1^ were assigned to *ν*(C–S) and *ν*(M–S), respectively [22, 25]. In the spectra of metal complexes, a broad band in the region 3200–3490 cm^−1^ indicated the presence of coordinated water molecules. A strong band in the region 1738–1750 cm^−1^ has been assigned to *ν*(OOCCH_3_) in 1 : 1 metal complexes.

### 3.2. ^1^H NMR Spectra

The ^1^H NMR spectra have been recorded for Schiff bases and Zn(II) complexes (Table 2). The NMR spectra of metal complexes indicated a shift of electron density from the ligand to metal. The ligands [HL^1-2^] showed characteristic azomethine proton singlet at *δ* 10.40 and 10.55 ppm, respectively. The characteristic signal, due to azomethine proton deshielded in the spectra of metal complexes, suggests coordination of metal ion to azomethine nitrogen atom [23, 25, 26]. In addition to this, the signal at *δ* 11.10–11.09 ppm is ascribed to SH proton. Disappearance of SH protons signal in the spectra of metal complexes supported the deprotonation of thiol group and chelation with metal (M–S). The aromatic protons of Schiff bases appeared as a double of doublet and two doublets at *δ* 7.35, 7.52 and 8.08 ppm, respectively. These aromatic protons signals show a slight downfield shift upon coordination. In the spectra of 1 : 1 Zn complexes singlet at *δ* 2.29 and 2.32 ppm are due to methyl group of acetate ion. 

### 3.3. Magnetic Measurements and Electronic Spectra

The magnetic moment measurements and electronic spectra provided good information regarding the arrangements of the ligands around the metal ions (Table 3). The 1 : 1 and 1 : 2 Co(II) complexes show magnetic moment (*μ*_eff._) 4.83–4.95 BM, well within the expected range of octahedral complex (4.3–5.2 BM) [24, 27]. Co(II) complexes exhibit two absorption bands in the region 10885–10935 cm^−1^ (*ν*_1_) and 20010–20998 cm^−1^ (*ν*_3_), which are assigned to ^4^T_1g_(F) → ^4^T_2g_(F) (*ν*_1_); ^4^T_1g_(F) → ^4^T_1g_(P) (*ν*_3_) transitions [23–25, 27]. These are the characteristic bands of high spin octahedral Co(II) complexes; *ν*_2_ is not observed, but it can be calculated [28, 29] by using relation *ν*_2_ = *ν*_1_ + 10Dq, which is very close to (*ν*_3_) transition. The ligand field parameters (Dq, B, *β*, *β*%) have also been calculated for Co(II) complexes by using Band-fitting equation [30]. The crystal field stabilizing energy (Dq) value was found to be ~1200 cm^−1^. These values are well within the range reported for the octahedral complexes [30]. The Racah parameter (B) is found to be 683–755 cm^−1^ (<971 cm^−1^), suggesting an overlapping of ligand metal orbital's. The nephelauxetic ratio (*β*) for the 1 : 1 and 1 : 2 cobalt complexes is less than one suggesting partial covalency in the metal ligand bond.

The nickel complexes show magnetic moment in the range 3.43–3.47 BM, which corresponds to octahedral environment around the central metal ion. Nickel(II) complexes generally show three absorption bands in octahedral environment corresponding to ^3^A_2g_(F) → ^3^T_2g_(F) (*ν*_1_), ^3^A_2g_(F) → ^3^T_1g_(F) (*ν*_2_) and ^3^A_2g_(F) → 3T_1g_(P) (*ν*_3_) transitions [28]. The 1 : 1 and 1 : 2 Ni(II) complexes of HL^1-2^ Schiff bases also show above three transitions in the region 9999–10123 cm^−1^ (*ν*_1_), 16380–17320 cm^−1^ (*ν*_2_), and 24885–24993 cm^−1^ (*ν*_3_), suggesting distorted octahedral geometry for the complexes. The ligand field parameters (Dq, B, *β*, *β*%) have also been calculated for Ni(II) complexes by using Band-fitting equation [30]. These parameters indicate significant covalent character of the metal ligand bonds.

The Cu(II) complex has magnetic moment value 1.92–2.11 BM, which fall in the normal range (1.7–2.2 BM). The electronic spectra of Cu(II) complexes showed broad band around 18550 cm^−1^, which is assigned to ^2^B_1g_ → ^2^A_1g_ (*ν*_1_) transition. It is a characteristic band of square planar geometry around the Cu(II) [31]. 

### 3.4. Thermal Studies

**Figure 2:**
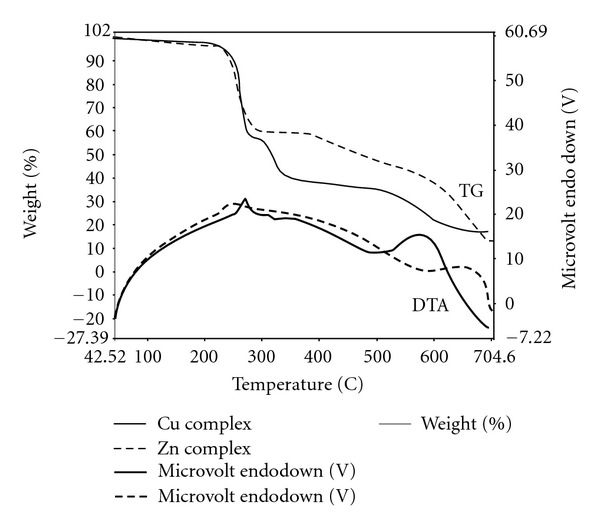
Thermal analyses curve of Zn(L^1^)_2_·2H_2_O and Cu(L^2^)_2_.

Thermogravimetric analysis was carried out for Cu(L^2^)_2_ and Zn(L^1^)_2_·2H_2_O (Figure 2) from 80°C to 700°C in atmospheric air [22, 23, 25]. The decomposition temperature, pyrolysed products, percentage mass loss of the complexes, and the ash (percent) are given in Table 4. The thermograms for both of these complexes show three decomposition steps. In case of Cu(L^2^)_2_ complex, the first step 50–305°C results in a mass loss of 45.29% (calcd. 47.90%) corresponding to a loss organic moiety. The 2nd and 3rd steps (305–695°C) correspond to removal of two triazole molecules with mass loss of 40.32% (calcd. 42.52%) of the ligand leaving metal oxide as residue.

In case of Zn(L^1^)_2_·2H_2_O complex, the first step 50–230°C results in a mass loss of 4.98% (calcd. 5.37%) corresponding to loss of two water molecules. The 2nd steps corresponds to loss of organic moiety with mass loss 51.50% (calcd. 51.30%) in the temperature range 230–560°C. The 3rd step (560–700°C) corresponds to removal of two triazole molecules with mass loss of 31.12% (calcd. 33.73%). The decomposition of both of the complexes ended with oxide formation.

### 3.5. ESR Spectra

ESR spectra of 1 : 1 and 1 : 2 copper(II) complexes in solid state at 298 K show an intense broad signal having no hyperfine structure. The observed g values for Cu(L^1^)(OAc)·H_2_O at room temperature are *g*_||_ = 2.18, *g*_⊥_ = 2.10, *g*_av_ = 2.12, *G* = 1.81 and for Cu(L^1^)_2_ at room temperature are *g*_||_ =2.19, *g*_⊥_ = 2.12, *g*_av_ = 2.14, *G* = 1.59. The trend in *g* values (*g*_||_ > *g*_⊥_ > 2.00) suggested that the unpaired electron is localized in the *dx* ^2^-*y* ^2^ orbital and corresponds to square planar geometry of the complexes. The *g*_||_ < 2.3 value confirms the covalent character of the metal ligand bond. The axial symmetry parameter *G* is less than 4.0, indicates considerable exchange interaction in the solid complex [32].

### 3.6. Antimicrobial Discussion

The Schiff bases and their metal complexes were screened for their antibacterial and antifungal activity. All the tested chemical compounds possessed variable antibacterial activity against *Staphylococcus aureus, Bacillus subtilis* and antifungal activity against *Aspergillus niger* and *Aspergillus flavus.* However the compounds in this series were not effective against any Gram-negative bacteria (*E. coli *and *P. aeruginosa*). Positive controls produced significantly sized inhibition zones against the tested bacteria and fungi; however, negative control produced no observable inhibitory effect against any of the test organism as shown in Tables 5, 6, and 7.

**Figure 3:**
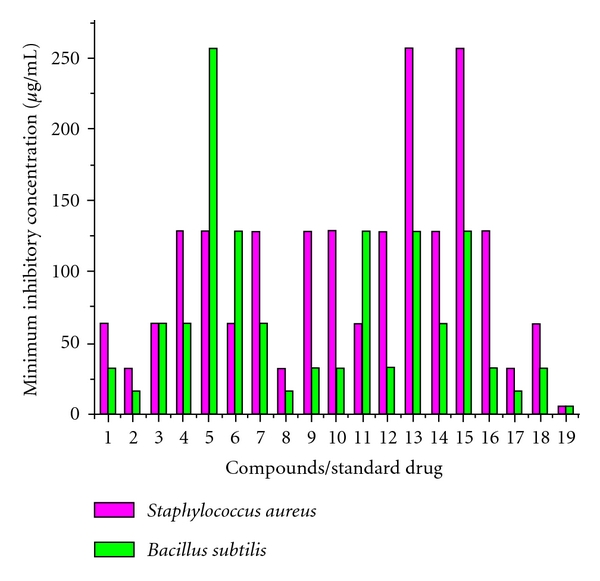
Comparison of minimum inhibitory concentration of compounds with Ciprofloxacin.

The tested chemical compounds showed zone of inhibition ranging between 15 and 26 mm against the Gram positive bacteria. On the basis of zone of inhibition produced against the test bacterium, Co(L^1^)(OAc)·3H_2_O and Zn(L^1^)(OAc)·3H_2_O were found to be most effective against *S. aureus* with zone of inhibition ranging between 23.6 mm and 20.6 mm, respectively, and four compounds HL^1^, Co(L^1^)(OAc)·3H_2_O, Zn(L^1^)(OAc)·3H_2_O and Zn(L^2^)(OAc)·3H_2_O were found to be best against *B. subtilis*, with zone of inhibition ranging between 21.6 mm and 23.6 mm (Table 5). In the whole series, the MIC of chemical compounds (Figure 3) ranged between 16 and 256 *μ*g/mL against Gram positive bacteria. Compound Co(L^1^)(OAc)·3H_2_O, Zn(L^1^)(OAc)·3H_2_O and Zn(L^2^)(OAc)·3H_2_O were found to be best, as they exhibit the lowest MIC of 32 *μ*g/mL against *S. aureus *and 16 *μ*g/mL against *B. subtilis* (Table 6).

All the synthesized compounds screened for their antifungal activity, among these three compounds HL^1^, Co(L^1^)(OAc)·3H_2_O, and Co(L^1^)_2_·2H_2_O showed more than 55% inhibition of mycelial growth against* Aspergillus niger,* whereas three compounds HL^1^, Co(L^1^)(OAc)·3H_2_O, and Zn(L^1^)(OAc)·3H_2_O, showed more than 55% inhibition of mycelial growth against* A. flavus. *Co(L^1^)(OAc)·3H_2_O showed the highest inhibition of fungal mycelium (61%) against *A. flavus *(Table 7). Among all the tested chemical compounds, Co(L^1^)(OAc)·3H_2_O showed highest antibacterial and antifungal activity. The antimicrobial studies suggested that the Schiff bases were found to be biologically active and their metal complexes showed significantly enhanced antibacterial and antifungal activity against microbial strains in comparison to the free ligands. The overtone's concept [33] and Tweedy's chelation theory [34] can be used to explain the enhanced in antimicrobial activity of the metal complexes. According to the Overtone's concept of cell permeability, the lipid membrane surrounding the cell favors the passage of only lipid-soluble materials; therefore, liposolubility is an important factor which controls the antimicrobial activity. On chelation, polarity of the metal ion is reduced to a greater extent due the overlapping of the ligand orbital and partial sharing of the positive charge of the metal ion with donor groups. Moreover, delocalization of the π-electrons over the whole chelate ring is increased and lipophilicity of the complexes is enhanced. The increased lipophilicity enhances the penetration of the complexes into the lipid membranes and blocks the metal binding sites in the enzymes of microorganisms. These complexes also disturb the respiration process of the cell and thus block the synthesis of proteins, which restricts further growth of the organism. In general, metal complexes are more active than ligands as they may serve as principal cytotoxic species. 

## 4. Conclusion

**Figure 4:**
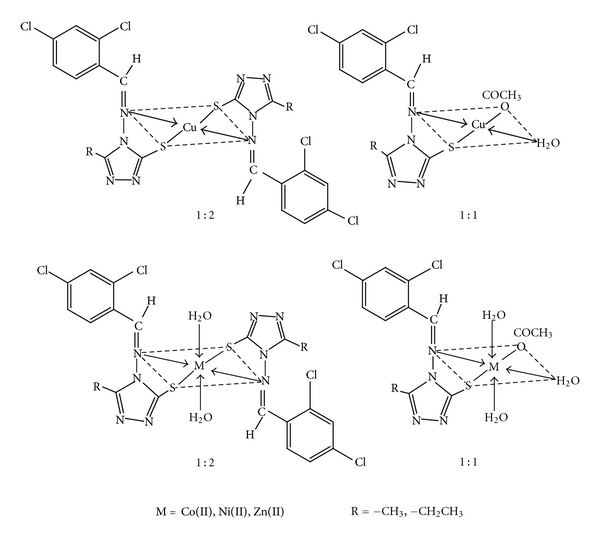
Structures of metal complexes.

The Schiff bases HL^1^ and HL^2^ coordinate in 1:1 and 1:2 metal-ligand ratios, as confirmed by analytical, IR, PMR, electronic, magnetic measurements, and thermal studies. The presence of coordinated water in metal complexes was confirmed by IR and TG studies. The trend in *g* values (*g*_||_ > *g*_⊥_ > 2.00) suggested that the unpaired electron is localized in the *dx* ^2^-*y* ^2^ orbital and corresponds to square planar geometry of the copper complexes. Based on these, the proposed structures are shown in Figure 4. The antimicrobial studies suggested that the Schiff bases were found to be biologically active, and their metal complexes showed significantly enhanced antibacterial and antifungal activity against microbial strains in comparison to the free ligands. Thus, exhibiting their broad spectrum nature can be further used in pharmaceutical industry for mankind, as an antimicrobial agent, after testing its toxicity to human beings.

## Figures and Tables

**Table 1:** IR spectral data of the ligands and their metal complexes (cm^−1^).

Compound	*ν*(N=CH)	*ν*(C–S)	*ν*(S–H)	*ν*(OOCCH_3_)	*ν*(H_2_O/OH)	*ν*(M–S)	*ν*(M–N)
HL^1^	1595	—	2732	—	—	—	—
Co(L^1^)(OAc)·3H_2_O	1585	741	—	1744	3426	338	498
Co(L^1^)_2_·2H_2_O	1585	743	—	—	3488	334	494
Ni(L^1^)(OAc)·3H_2_O	1587	749	—	1742	3471	342	510
Ni(L^1^)_2_·2H_2_O	1588	733	—	—	3394	339	514
Cu(L^1^)(OAc)·H_2_O	1590	748	—	1744	3310	345	513
Cu(L^1^)_2_	1589	750	—	—	—	348	491
Zn(L^1^)(OAc)·3H_2_O	1588	745	—	1750	3441	352	489
Zn(L^1^)_2_·2H_2_O	1585	748	—	—	3440	348	492
HL^2^	1597	—	2703	—	—	—	—
Co(L^2^)(OAc)·3H_2_O	1585	733	—	1738	3294	344	485
Co(L^2^)_2_·2H_2_O	1586	741	—	—	3310	338	488
Ni(L^2^)(OAc) ·3H_2_O	1585	771	—	1744	3280	338	489
Ni(L^2^)_2_·2H_2_O	1587	755	—	—	3287	335	492
Cu(L^2^)(OAc)·H_2_O	1585	733	—	1746	3400	343	503
Cu(L^2^)_2_	1586	733	—	—	—	348	503
Zn(L^2^)(OAc)·3H_2_O	1589	748	—	1740	3318	347	500
Zn(L^2^)_2_·2H_2_O	1589	771	—	—	3310	349	497

**Table 2:** ^1^H NMR spectral data of Schiff bases and their metal complexes.

Compounds	^1^H NMR (CDCl_3_/DMSO-d_6_) (ppm)
HL^1^ [C_10_H_8_Cl_2_N_4_S]	2.49 (s, 3H, –CH_3_), 7.35 (dd, 1H, Ar–H), 7.52 (d, 1H, Ar H), 8.08 (d, 1H, ArH), 10.40 (s, 1H, –N=CH–), 11.10 (s, 1H, –SH)
Zn(L^1^)OAc·3H_2_O [C_12_H_16_Cl_2_N_4_O_5_SZn]	2.37 (s, 3H, –CH_3_), 7.41 (dd, 1H, Ar–H), 7.79 (d, 1H, Ar–H), 8.12 (d, 1H, Ar–H), 10.52 (s, 1H, –N=CH–), 2.29 (s, 3H, CH_3_COO)
Zn(L^1^)_2_·2H_2_O [C_20_H_18_Cl_4_N_8_O_2_S_2_Zn]	2.58 (s, 3H, –CH_3_), 7.47 (dd, 1H, Ar–H), 7.78 (d, 1H, Ar–H), 8.08 (d, 1H, Ar–H), 10.58(s, 1H, –N=CH–)
HL^2^ [C_11_H_10_Cl_2_N_4_S]	2.84 (q, 2H, –CH_2_CH_3_), 1.35 (t, 3H, –CH_2_CH_3_), 7.35 (dd, 1H, Ar–H), 7.52 (d, 1H, Ar–H), 8.07 (d, 1H, Ar–H), 10.55 (s, 1H, –N=CH–), 11.09 (s, 1H, –SH)
Zn(L^2^)OAc·3H_2_O [C_13_H_18_Cl_2_N_4_O_5_SZn]	2.67 (q, 2H, –CH_2_CH_3_), 1.22 (t, 3H, –CH_2_CH_3_), 7.43 (dd, 1H, Ar–H), 7.82 (d, 1H, Ar–H), 8.13 (d, 1H, Ar H), 10.61 (s, 1H, –N=CH–), 2.32 (s, 3H, CH_3_COO)
Zn(L^2^)_2_·2H_2_O [C_22_H_22_Cl_4_N_8_O_2_S_2_Zn]	2.66 (q, 2H, –CH_2_CH_3_), 1.18 (t, 3H, –CH_2_CH_3_), 7.55 (dd, 1H, Ar–H), 7.83 (d, 1H, Ar–H), 8.13 (d, 1H, Ar H), 10.60 (s, 1H, –N=CH–)

**Table 3:** Electronic spectral data of metal complexes.

Compound	Transitions (cm^−1^)			Dq cm^−1^	B cm^−1^	*ν*_2_/*ν*_1_	*β*	*β*%
	*ν*_1_	*ν*_2_	*ν*_3_					
Co(L^1^)(OAc)·3H_2_O	10897	23031*	20998	1213.4	755.9	2.11	0.778	22.2
Co(L^1^)_2_·2H_2_O	10885	22884*	20993	1199.9	748.1	2.10	0.770	23.0
Ni(L^1^)(OAc)·3H_2_O	10117	17201	24932	1011.7	785.4	1.70	0.754	24.5
Ni(L^1^)_2_·2H_2_O	10123	17320	24950	1012.3	793.4	1.71	0.762	23.7
Co(L^2^)(OAc)·3H_2_O	10923	23017*	20010	1209.4	683.9	2.09	0.704	29.6
Co(L^2^)_2_·2H_2_O	10935	23041*	20016	1210.6	683.5	2.10	0.703	29.6
Ni(L^2^)(OAc)·3H_2_O	9999	16380	24885	999.9	751.2	1.63	0.721	27.9
Ni(L^2^)_2_·2H_2_O	9957	16397	24889	995.7	761.0	1.64	0.731	26.9

*Calculated value.

**Table 4:** Thermoanalytical results (TG) of the metal complexes.

Compound	Steps	Temp. (°C)	TG mass%		Assignments
			Calcd.	Found	
Cu(L^2^)_2_	1st 2nd 3rd	80–305 305–505 505–695	47.90 21.26 21.26 11.98	45.29 20.27 20.05 15.16	−C_14_H_8_Cl_4_ (organic moiety) −C_4_H_5_N_4_S (triazole ring) −C_4_H_5_N_4_S (triazole ring) −CuO (residue)
Zn(L^1^)_2_·2H_2_O	1st 2nd 3rd	80–230 230–560 560–700	5.37 51.30 33.73 11.90	4.98 51.50 31.12 12.40	−H_4_O_2_ (two water molecule) −C_14_H_8_Cl_4_N_2_ (organic moiety) −C_6_H_6_N_6_S_2_ (triazole ring) −ZnO (residue)

**Table 5:** Antibacterial activity of synthesized compounds.

Compound	Diameter of growth of inhibition zone	
	(mm)^a^	
	*Staphylococcus aureus*	*Bacillus subtilis*
HL^1^	19.0	21.3
Co(L^1^)(OAc)·3H_2_O	23.6	25.6
Co(L^1^)_2_·2H_2_O	16.6	19.6
Ni(L^1^)(OAc)·3H_2_O	16.3	18.3
Ni(L^1^)_2_·2H_2_O	14.6	19.6
Cu(L^1^)(OAc)·H_2_O	20.3	18.3
Cu(L^1^)_2_	15.6	17.0
Zn(L^1^)(OAc)·3H_2_O	20.6	23.6
Zn(L^1^)_2_·2H_2_O	19.3	20.6
HL^2^	17.3	18.3
Co(L^2^)(OAc)·3H_2_O	16.6	19.3
Co(L^2^)_2_·2H_2_O	17.6	20.6
Ni(L^2^)(OAc)·3H_2_O	15.6	18.3
Ni(L^2^)_2_·2H_2_O	15.0	16.6
Cu(L^2^)(OAc)·H_2_O	16.3	18.5
Cu(L^2^)_2_	19.6	21.2
Zn(L^2^)(OAc)·3H_2_O	20.3	21.6
Zn(L^2^)_2_·2H_2_O	18.3	19.6
Ciprofloxacin	26.6	24.0

^a^Values, including diameter of the well (8 mm), are means of three replicates.

**Table 6:** Minimum inhibitory concentration (MIC) (in *μ*g/mL) of compounds.

Sr. No.	Compound	*Staphylococcus aureus*	*Bacillus subtilis*
1	HL^1^	64	32
2	Co(L^1^)(OAc)·3H_2_O	32	16
3	Co(L^1^)_2_·2H_2_O	64	64
4	Ni(L^1^)(OAc)·3H_2_O	128	64
5	Ni(L^1^)_2_·2H_2_O	128	256
6	Cu(L^1^)(OAc)·H_2_O	64	128
7	Cu(L^1^)_2_	128	64
8	Zn(L^1^)(OAc)·3H_2_O	32	16
9	Zn(L^1^)_2_·2H_2_O	128	32
10	HL^2^	128	32
11	Co(L^2^)(OAc)·3H_2_O	64	128
12	Co(L^2^)_2_·2H_2_O	128	32
13	Ni(L^2^)(OAc)·3H_2_O	256	128
14	Ni(L^2^)_2_·2H_2_O	128	64
15	Cu(L^2^)(OAc)·H_2_O	256	128
16	Cu(L^2^)_2_	128	32
17	Zn(L^2^)(OAc)·3H_2_O	32	16
18	Zn(L^2^)_2_·2H_2_O	64	32
19	Ciprofloxacin	5	5

**Table 7:** Antifungal activity of synthesized compounds.

Compound	Mycelial growth inhibition (%)	
	*Aspergillus niger*	*Aspergillus flavus*
HL^1^	57.7	58.8
Co(L^1^)(OAc)·3H_2_O	58.8	61.1
Co(L^1^)_2_·2H_2_O	56.6	55.5
Ni(L^1^)(OAc)·3H_2_O	53.3	55.5
Ni(L^1^)_2_·2H_2_O	51.1	47.7
Cu(L^1^)(OAc)·H_2_O	50.0	48.8
Cu(L^1^)_2_	53.3	52.5
Zn(L^1^)(OAc)·3H_2_O	54.4	56.6
Zn(L^1^)_2_·2H_2_O	51.1	50.0
HL^2^	48.8	45.5
Co(L^2^)(OAc)·3H_2_O	46.6	47.7
Co(L^2^)_2_·2H_2_O	48.8	50.0
Ni(L^2^)(OAc)·3H_2_O	44.4	45.5
Ni(L^2^)_2_·2H_2_O	45.5	43.3
Cu(L^2^)(OAc)·H_2_O	48.8	50.0
Cu(L^2^)_2_	52.5	54.4
Zn(L^2^)(OAc)·3H_2_O	53.3	52.5
Zn(L^2^)_2_·2H_2_O	50.0	51.1
Fluconazole	81.1	77.7

## References

[1] Chohan ZH, Supuran CT (2005). In-vitro antibacterial and cytotoxic activity of cobalt (II), copper (II), nickel (II) and zinc (II) complexes of the antibiotic drug cephalothin (keflin). *Journal of Enzyme Inhibition and Medicinal Chemistry*.

[2] Singh K, Kumar Y, Pundir RK (2010). Synthesis and characterization of biologically active organosilicon(IV) complexes with Schiff bases derived from o-aminothiophenol. *Synthesis and Reactivity in Inorganic, Metal-Organic and Nano-Metal Chemistry*.

[3] Altundas A, Nursen S, Colak N, Ogutchi H (2010). Synthesis and biological activity of new cycloalkylthiophene-Schiff bases and their Cr(III) and Zn(II) complexes. *Medicinal Chemistry Research*.

[4] Garnovskii AD, Vasilchenko IS, Garnovskii DA, Kharisov BI (2009). Molecular design of mononuclear complexes of acyclic Schiff-base ligands. *Journal of Coordination Chemistry*.

[5] Heshmatpour F, Ghassemzadeh M, Bahemmat S, Malakootikhah J, Neumüller B, Rothenberger A (2007). Synthesis, characterization and molecular structure of a new tetrameric palladium(II) complex containing schiff-bases derived from AMTTO (AMTTO = 4-amino-6-methyl-1,2,4-triazine-thione-5-one). *Zeitschrift fur Anorganische und Allgemeine Chemie*.

[6] Nuria A, Cabeza I, Rosario A, Alvarez VY, Akdi K, Carretero M (2005). Synthesis, structure and biological activity of a new and efficient Cd(II)–uracil derivative complex system for cleavage of DNA. *Journal of Biological Inorganic Chemistry*.

[7] Turan-Zitouni G, Kaplancikli ZA, Yildiz MT, Chevallet P, Kaya D (2005). Synthesis and antimicrobial activity of 4-phenyl/cyclohexyl-5-(1- phenoxyethyl)-3-[*N*-(2-thiazolyl)acetamido]thio-4H-1,2,4-triazole derivatives. *European Journal of Medicinal Chemistry*.

[8] Walczak K, Gondela A, Suwin’ ski J (2004). Synthesis and anti-tuberculosis activity of N-aryl-C-nitroazoles. *European Journal of Medicinal Chemistry*.

[9] Mavrova AT, Wesselinova D, Tsenov YA, Denkova P (2009). Synthesis, cytotoxicity and effects of some 1,2,4-triazole and 1,3,4-thiadiazole derivatives on immunocompetent cells. *European Journal of Medicinal Chemistry*.

[10] Al-Soud YA, Al-Masoudi NA, Ferwanah AERS (2003). Synthesis and properties of new substituted 1,2,4-triazoles: potential antitumor agents. *Bioorganic and Medicinal Chemistry*.

[11] Almasirad A, Tabatabai SA, Faizi M (2004). Synthesis and anticonvulsant activity of new 2-substituted-5-[2-(2- fluorophenoxy)phenyl]-1,3,4-oxadiazoles and 1,2,4-triazoles. *Bioorganic and Medicinal Chemistry Letters*.

[12] Amir M, Shikha K (2004). Synthesis and anti-inflammatory, analgesic, ulcerogenic and lipid peroxidation activities of some new 2-[(2,6-dichloroanilino) phenyl]acetic acid derivatives. *European Journal of Medicinal Chemistry*.

[13] Brandt CD, Kitchen JA, Beckmann U, White NG, Jameson GB, Brooker S (2007). Synthesis and structures of 3,5-disubstituted 1,2,4-triazole head units and incorporation of 3,5-dibenzoyl-1,2,4-triazolate into new [2 + 2] Schiff-base macrocyclic complexes. *Supramolecular Chemistry*.

[14] Bekircan O, Bektas H (2006). Synthesis of new bis-1,2,4-triazole derivatives. *Molecules*.

[15] Vogel AI (1999). *A Text Book of Quantitative Chemical Analysis*.

[16] Bala S, Gupta RP, Sachdeva ML, Singh A, Pujari HK (1978). Heterocyclic systems containing bridgehead nitrogen atom: part XXXIII—synthesis of s-Triazolo [3,4-b][1,3,4] thiadiazine, s-triazolo-[3,4-b][1,3,4]thiadiazino[6,7-b] quinoxaline & as-triazino-[3,4-b] [1,3,4] thiadiazines. *Indian Journal of Chemistry*.

[17] Aneja KR, Sharma C, Joshi R (2010). Fungal infection of the ear: a common problem in the north eastern part of Haryana. *International Journal of Pediatric Otorhinolaryngology*.

[18] Ahmad I, Beg AZ (2001). Antimicrobial and phytochemical studies on 45 Indian medicinal plants against multi-drug resistant human pathogens. *Journal of Ethnopharmacology*.

[19] Andrews JM (2001). Determination of minimum inhibitory concentrations. *Journal of Antimicrobial Chemotherapy*.

[20] Okeke MI, Iroegbu CU, Eze EN, Okoli AS, Esimone CO (2001). Evaluation of extracts of the root of Landolphia owerrience for antibacterial activity. *Journal of Ethnopharmacology*.

[21] Al-Burtamani SKS, Fatope MO, Marwah RG, Onifade AK, Al-Saidi SH (2005). Chemical composition, antibacterial and antifungal activities of the essential oil of Haplophyllum tuberculatum from Oman. *Journal of Ethnopharmacology*.

[22] Singh K, Singh DP, Barwa MS, Tyagi P, Mirza Y (2006). Antibacterial Co(II), Ni(II), Cu(II) and Zn(II) complexes of Schiff bases derived from fluorobenzaldehyde and triazoles. *Journal of Enzyme Inhibition and Medicinal Chemistry*.

[23] Singh K, Barwa MS, Tyagi P (2006). Synthesis, characterization and biological studies of Co(II), Ni(II), Cu(II) and Zn(II) complexes with bidentate Schiff bases derived by heterocyclic ketone. *European Journal of Medicinal Chemistry*.

[24] Kulkarni AD, Patil SA, Naik VH, Badami PS (2011). DNA cleavage and antimicrobial investigation of Co(II), Ni(II), and Cu(II) complexes with triazole schiff base: synthesis and spectral characterization. *Medicinal Chemistry Research*.

[25] Singh K, Singh DP, Barwa MS, Tyagi P, Mirza Y (2006). Some bivalent metal complexes of Schiff bases containing N and S donor atoms. *Journal of Enzyme Inhibition and Medicinal Chemistry*.

[26] Bagihalli GB, Patil SA, Badami PS (2009). Synthesis, physicochemical investigation and biological studies of zinc(II) complexes with 1,2,4-triazole schiff bases. *Journal of the Iranian Chemical Society*.

[27] Avaji PG, Patil SA, Badami PS (2008). Synthesis, spectral, thermal, solid state d.c. electrical conductivity and biological studies of Co(II), Ni(II) and Cu(II) complexes with 3-substituted-4-amino (indole-3-aldehydo)-5-mercapto-1,2,4-triazole Schiff bases. *Journal of Coordination Chemistry*.

[28] Cotton FA, Williknson G, Murillo CA, Bochman M (2003). *Advanced Inorganic Chemistry*.

[29] Patra AK, Dhar S, Nethaji M, Chakravarty AR (2005). Metal-assisted red light-induced DNA cleavage by ternary L-methionine copper(II) complexes of planar heterocyclic bases. *Dalton Transactions*.

[30] Lever ABP (1968). *Inorganic Spectroscopy*.

[31] Osman AH, Saleh MS, Sanaa MM (2004). Synthesis, characterization, and photochemical studies of some copper complexes of schiff bases derived from 3-hydrazino-6-methyl[1,2,4]triazin-5(4*H*) one. *Synthesis and Reactivity in Inorganic and Metal-Organic Chemistry*.

[32] Singh OI, Damayanti M, Singh NR, Singh RKH, Mohapatra M, Kadam RM (2005). Synthesis, EPR and biological activities of bis(1-n-butylamidino-O-alkylurea)copper(II)chloride complexes: EPR evidence for binuclear complexes in frozen DMF solution. *Polyhedron*.

[33] Anjaneyulu Y, Rao RP (1986). Preparation, characterization and antimicrobial activity studies on some ternary complexes of Cu(II) with acetyl acetone and various salicylic acids. *Synthesis and Reactivity in Inorganic, Metal-Organic, and Nano-Metal Chemistry*.

[34] Dharmaraj N, Viswanathamurthi P, Natarajan K (2001). Ruthenium(II) complexes containing bidentate Schiff bases and their antifungal activity. *Transition Metal Chemistry*.

